# Cardiac patients’ surgery outcome and associated factors in Ethiopia: application of machine learning

**DOI:** 10.1186/s12887-024-04870-4

**Published:** 2024-06-18

**Authors:** Melaku Tadege, Awoke Seyoum Tegegne, Zelalem G. Dessie

**Affiliations:** 1 College of Science, Bahir Dar University, Bahir Dar, Ethiopia; 2 Department of Statistics, Injibara University, Injibara, Amhara, Ethiopia; 3 Regional Data Management Center for Health (RDMC), Amhara Public Health Institute (APHI), Bahir Dar, Ethiopia; 4 School of Mathematics, Statistics and Computer Science, University of KwaZulu-Natal, Durban, South Africa

**Keywords:** Machine learning, Cardiac disease, Ethiopia, Cardiac surgery

## Abstract

**Introduction:**

Cardiovascular diseases are a class of heart and blood vessel-related illnesses. In Sub-Saharan Africa, including Ethiopia, preventable heart disease continues to be a significant factor, contrasting with its presence in developed nations. Therefore, the objective of the study was to assess the prevalence of death due to cardiac disease and its risk factors among heart patients in Ethiopia.

**Methods:**

The current investigation included all cardiac patients who had cardiac surgery in the country between 2012 and 2023. A total of 1520 individuals were participated in the study. Data collection took place between February 2022 and January 2023. The study design was a retrospective cohort since the study track back patients’ chart since 2012. Machine learning algorithms were applied for data analysis. For machine learning algorithms comparison, lift and AUC was applied.

**Results:**

From all possible algorithms, logistic algorithm at 90%/10% was the best fit since it produces the maximum AUC value. In addition, based on the lift value of 3.33, it can be concluded that the logistic regression algorithm was performing well and providing substantial improvement over random selection. From the logistic regression machine learning algorithms, age, saturated oxygen, ejection fraction, duration of cardiac center stays after surgery, waiting time to surgery, hemoglobin, and creatinine were significant predictors of death.

**Conclusion:**

Some of the predictors for the death of cardiac disease patients are identified as such special attention should be given to aged patients, for patients waiting for long periods of time to get surgery, lower saturated oxygen, higher creatinine value, lower ejection fraction and for patients with lower hemoglobin values.

## Background

Based on the WHO definition, cardiovascular diseases are a group of disorders of the heart and blood vessels. Cardiovascular disease is one of the leading responsible causes of death. The global burden of disease studies reported that there were about 422.7 million cases of cardiovascular disease, and 17.92 million died since 2015. Over the last two decades, the global disease trend has shifted from communicable to non-communicable diseases [1, 2]. According to World Health Organization statistics, 17.6 million people have died from cardiovascular disease (CVD) globally in 2012, accounting for 31 per cent of global deaths [3].

In low-income countries, living in poverty is highly affected by cardiovascular disease(CVD) [4]. Unlike developed countries [5], it remains main cause of preventable cardiac disease in the Sub-Saharan Africa, due to educational, environmental, and economic disadvantages and reduced access to health care [6]. In North West Ethiopia, from all cardiovascular disease assessments, nearly half $\left(49\text{\%}\right)$ of the study participants had valvular heart disease [7].

Current epidemiological data on valvular heart disease is critical for better understanding this evolving disease spectrum and allocating healthcare resources accordingly [8]. Epidemiological research on acquired and congenital valvular heart disease is limited. Changing the global trend of valvular heart disease is being examined due to ageing and advances in treatments, which is critical for advances in clinical practice and the preparation of health care policy. Because of ageing, the global prevalence of heart disease is increasing [9].

In Ethiopian, it is time for effective population-based, preventive health strategies for primary and secondary prevention of established cardiovascular disorders and risk factors [10]. Ethiopian population has seen major epidemiological shift. The main causes of death and illness change from infectious to non-communicable diseases [11]. One of the significant causes of morbidity and mortality is cardiovascular disease. However, there are no productive novel solutions to lower cardiovascular risk because there is a mistaken imagination that cardiovascular disease is solely a problem of the rich and developed world. There is also a tendency to associate cardiovascular mainly with hyper-nutrition [12, 13]. Cardiac disease is neglected by politicians and health related stakeholders [14].

Sufficient report on cardiovascular disorder is generally scarce in developing countries, including Ethiopia. According to a study in Tikur Anbessa and St.Paul’s Hospitals, cardiovascular diseases were one of the main causes of mortality and the most common reasons for hospitalization [15]. Evidence from cardiac center Ethiopia stated that cardiac disease patients increase dramatically from time to time. Another evidence from ministry of health shows, more than 40,000 cardiac disease patients have been waiting for cardiac surgery in Ethiopia since 2020. A report from cardiac center Ethiopia, around 7000 cardiac disease patients seek cardiac surgery at the waiting list [16].

Preventive treatment at an early stage of the disease is widely accepted to reduce morbidity and mortality. The benefits of early surgery should be balanced against the surgical risks and long-term outcomes. There is no adequate evidence on cardiac disease, specifically after cardiac surgery in Ethiopia. As such the study will serve as to deliver update information on cardiac patients’ status after surgery. In addition, this study aimed to compare the best machine learning algorithms for cardiac surgery outcomes and to identify the responsible predictors of death outcome.

## Participants and methods

### Study area and period

**Fig. 1:**
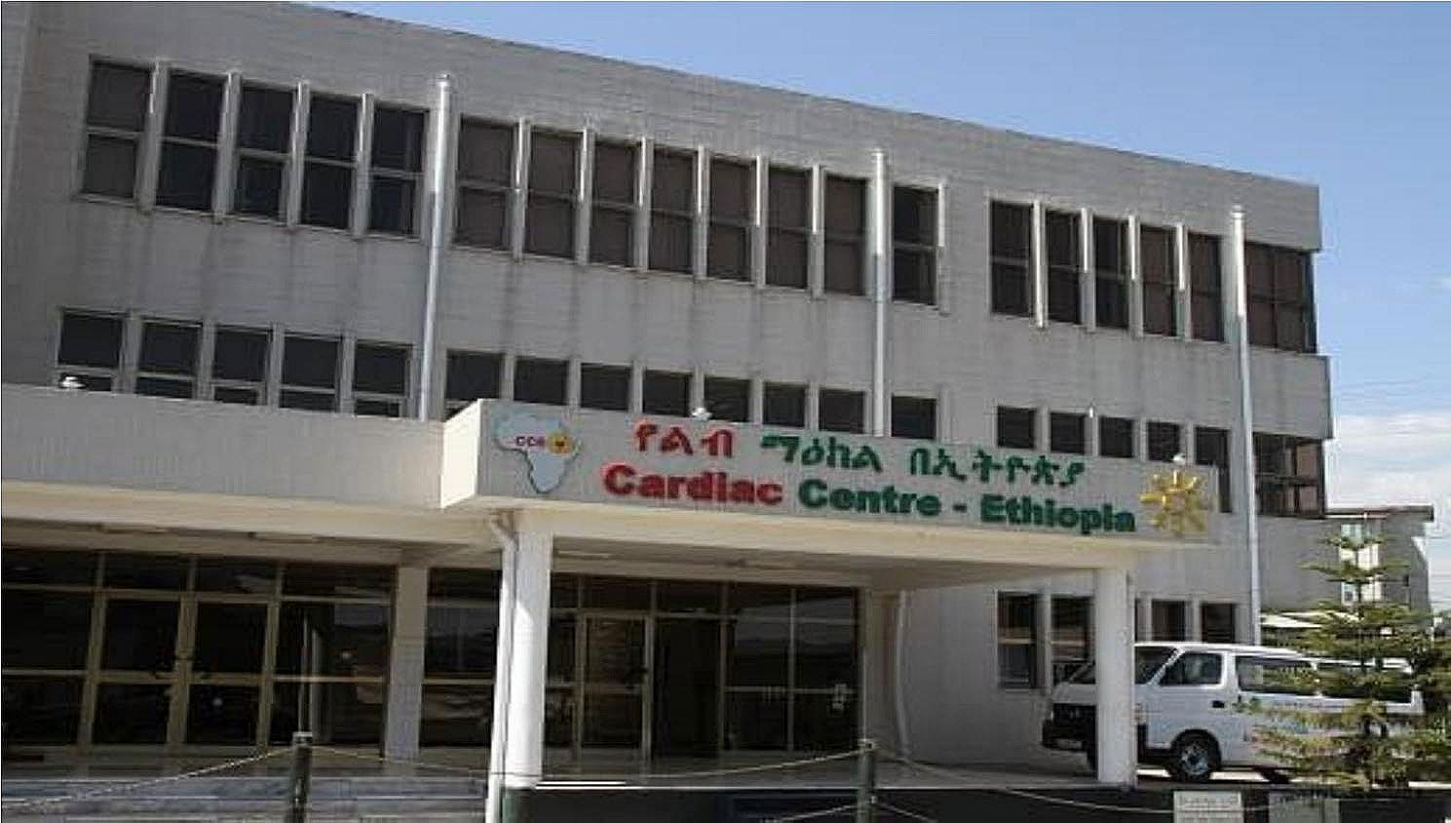
Cardiac center in Ethiopia

The study was done at two cardiac centers named as “Cardiac Center Ethiopia” and “Elouzeir Cardiac Center”. Those cardiac centers are located in Adis Ababa, the capital city of Ethiopia. Cardiac center Ethiopia was established in 2009 where as Elouzeir Cardiac Center was established in 2016. There are 15 cardiothoracic surgeons, from all, five can do open-heart surgery, one specialist might serve at more than one center. In cardiac center Ethiopia, more than 7000 patients were under waiting list to get the cardiac surgery service. Sine establishment, 1520 cardiac patients had surgery at those centers. Cardiac Center Ethiopia, currently provides the largest cardiac surgery in Ethiopia. This surgical center started to provide surgery service for patients with cardiac disease since 2009. A study was performed from 2012 to 2023 through utilizing patients’ follow-up charts. One of the study areas, called “cardiac center Ethiopia” (Fig. 1).

#### Study design:

The research was conducted using a retrospective cohort study design, focusing on a population of cardiac patients in Ethiopia who had surgery and follow-up appointments between 2012 and 2023.

#### Study population:

The study population was cardiac patients who had heart surgery from 2012 to 2023 in Ethiopia. In the study period, there are two cardiac centers in Ethiopia which serve as a fully functional cardiac surgery. Both cardiac centers were part of the investigation. In those centers, 1520 patients had cardiac surgery service. All 1520 patients were part of the study. Regarding inclusion criteria, all cardiac patients who have had surgery since 2012 was included.

#### Data Collection technique:

The information was gathered by utilizing structured questionnaires on an online data collection platform called Kobo Toolbox. The questionnaire was prepared in English language as of the chart form. The questionnaire was assessed in randomly selected patients’ charts; as such, pre-test was performed to check data quality. Health professionals were participated in the data collection journey. The data collection involved the participation of BSc nurses and medical doctors. In Ethiopia, there is a standardized patient chart form, developed according to the World Health Organization (WHO) standards, is universally used. Due to the absence of computerized data management systems, the data collectors manually retrieved patient information from the patients’ charts. The data collection process commenced after obtaining ethical clearance from Bahir Dar University Science College, and permission from the relevant healthcare provider institutions.

### Outcome variables

#### Death status:

The outcome variable for a cardiac patient’s possible outcome (Death, Alive) after surgery.

#### The predictor variables:

**Fig. 2:**
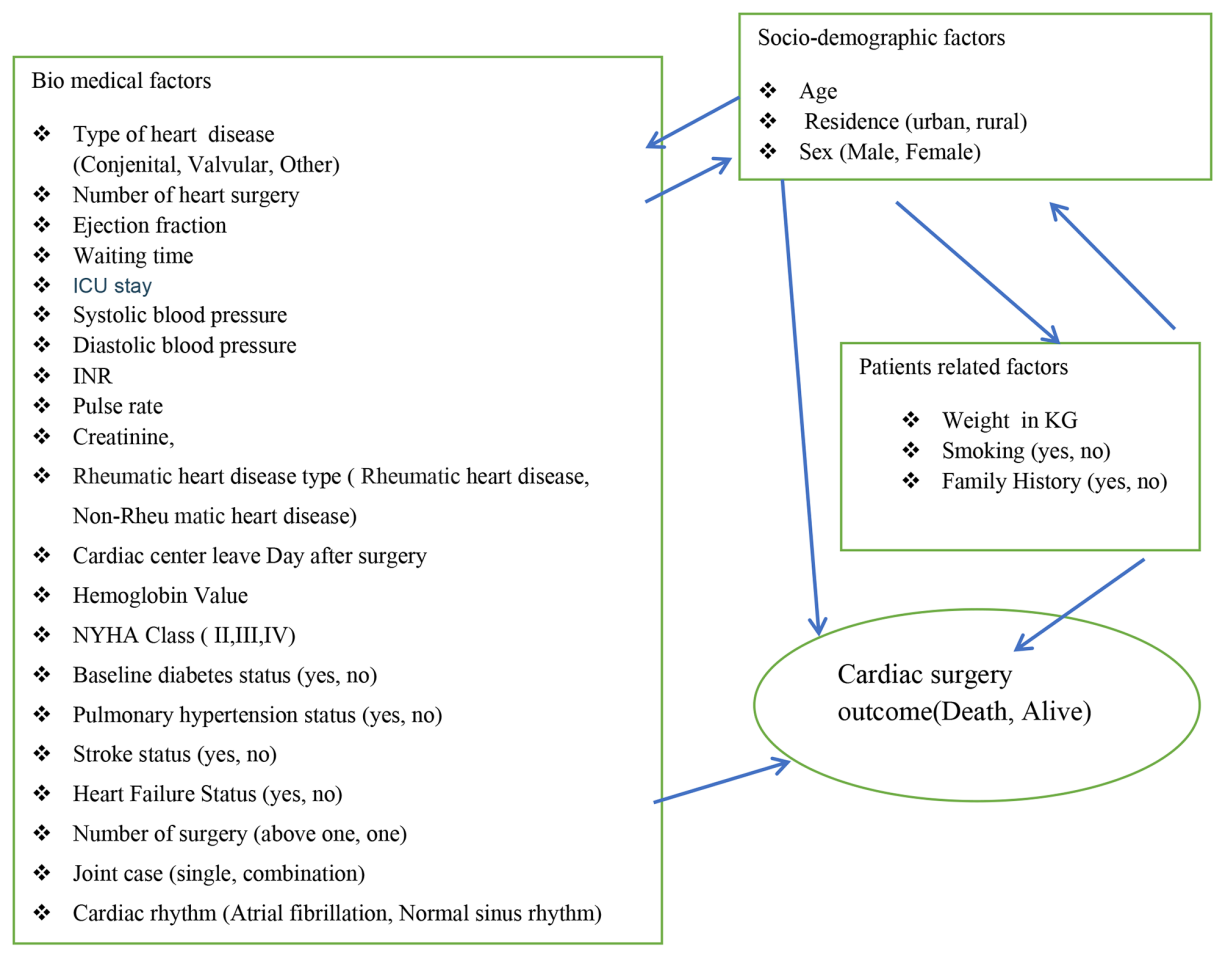
Conceptual framework

The predictor variables are summarized in as the following (Fig. 2).

### Data processing and analysis

Once the data collection process is complete, the collected data exported into Python and SPSS. Descriptive information characterises the study population. Categorical variables were expressed in terms of frequency and percentage whereas continuous variables were described in mean, range and standard deviation. Chi-square test of association was performed. Nine different machine learning algorithms were considered for comparison and analysis. Feature selection and analysis were performed using Python, IBM SPSS Modeler and IBM SPSS Statistics software.

### Machine learning

Machine learning is the technology that allows computers to create algorithms that can mimic human intelligence. Ideas inspire it in artificial intelligence, probability and statistics, computer science, information theory, control theory, psychology, and philosophy [17, 18]. This technology is used in various fields, including biomedical and medical applications [19, 20]. The most important feature of machine learning algorithms is their unique opportunity to predict and to understand the environment from input data [17]]. Machine learning is classified into three types based on the nature of the data labelling: supervised, unsupervised, and semi-supervised. Supervised learning derives an unknown output and input, mapping from the known output and input samples, with the output labelled classification [21]. The study used nine supervised machine learning algorithms for analysis and comparison. The following are the algorithms:

### Bayesian network

The Bayesian Network node allows you to create a probability model by combining observed and recorded evidence with real-world knowledge to determine the likelihood of events occurring. The node focuses on classification networks such as Tree Augmented Nave Bayes and Markov Blanket.

### C5

The C5.0 node constructs a decision tree or a rule set. The model divides the sample based on the field that provides the greatest information gain at each level. The target field must be categorical in nature.

### CHAID

The CHAID node generates decision trees using chi-square statistics to identify optimal splits. In contrast to the C&R Tree and QUEST nodes, CHAID can generate non binary trees, which means that some divisions have more than two branches. The target and input fields can be either continuous numeric ranges or categorical. Exhaustive CHAID is a CHAID modification that examines all possible splits more thoroughly but takes longer to compute.

### QUEST

The QUEST shows a binary classifier method for building decision trees, which is intended to reduce the processing time required for large classification and regression tree assessments while reducing the tendency found in classification tree methods to favour inputs with more splits. The input fields can be continuous numerical ranges, but the target field must be categorical.

### C&R tree

Regression and classification tree nodes generate a decision tree that can be used to predict or categorise future observations. The method divides the training records into segments by reducing impurity on every step, with a node in the tree considered “pure” if all of the cases in the node drop into a particular category of the target field. All splits are binary, with only two subgroups; target and input fields can be numeric ranges or categorical as nominal, ordinal, or flags.

### Decision list

The Decision Node List identifies groups or segments that show more or less a given binary value compared to the population. A list of decision models consists of a list of rules, where each rule has an event and a consequence. The rules are applied sequentially, with the first matching rule determining the outcome.

### Logistic

Logistic regression is a statistical technique used to classify data based on the value of an input field. It is similar to linear regression but uses a categorical rather than numerical purpose. Logistic regression is an example of a model commonly used in classification problems. The model includes a maximum likelihood estimation technique to estimate the default.

### Neural network

A neural network is a machine learning algorithm consisting of a set of nodes, the most important of which are the input layer [22], the hidden layer, and the output layer. It has a set of input neurons (X) that represent the data extracted from each feature in the data. Backpropagation is a technique used in neural networks, where the nonpredictive neural network is iterated before the weights of each neural connection are changed until there is less error [23, 24].

### Discriminant

Discriminant testing creates a predictive model for group membership. The model has a discriminating function or discriminating function for more than two groups that provides the best discrimination between groups based on a combination of different predictors. This study consists of a sample of experienced members; these functions can be used for new situations with parameter values ​​, but group membership is unknown.

#### Variable selection:

In the research, a feature selection method was employed to determine the significance of inputs in relation to a specific target. Out of all the predictor variables, the model incorporated a total of 12 variables.

### Model comparison

When compared to traditional models, machine learning models outperform them in classification problems. Raw data is rarely found in the form and shape required for machine learning algorithms to perform optimally. Because the algorithms used in machine learning are only numerical values, it is necessary to convert categorical variables to numerical values. As a result, the most important aspect of machine learning model applications is the pre-processing step [25–27]. The dataset’s categorical features are encoded to convert them into numerical values, and the continuous data in this study were normalised. For machine learning approaches, the dataset is randomly divided into two parts: a training dataset that trains the model and a test dataset that predicts the response variable and checks whether the predicted outcome is similar to the actual outcomes, and the validation dataset is used to estimate the parameters that will be used in the training models [28–30].

**Fig. 3:**
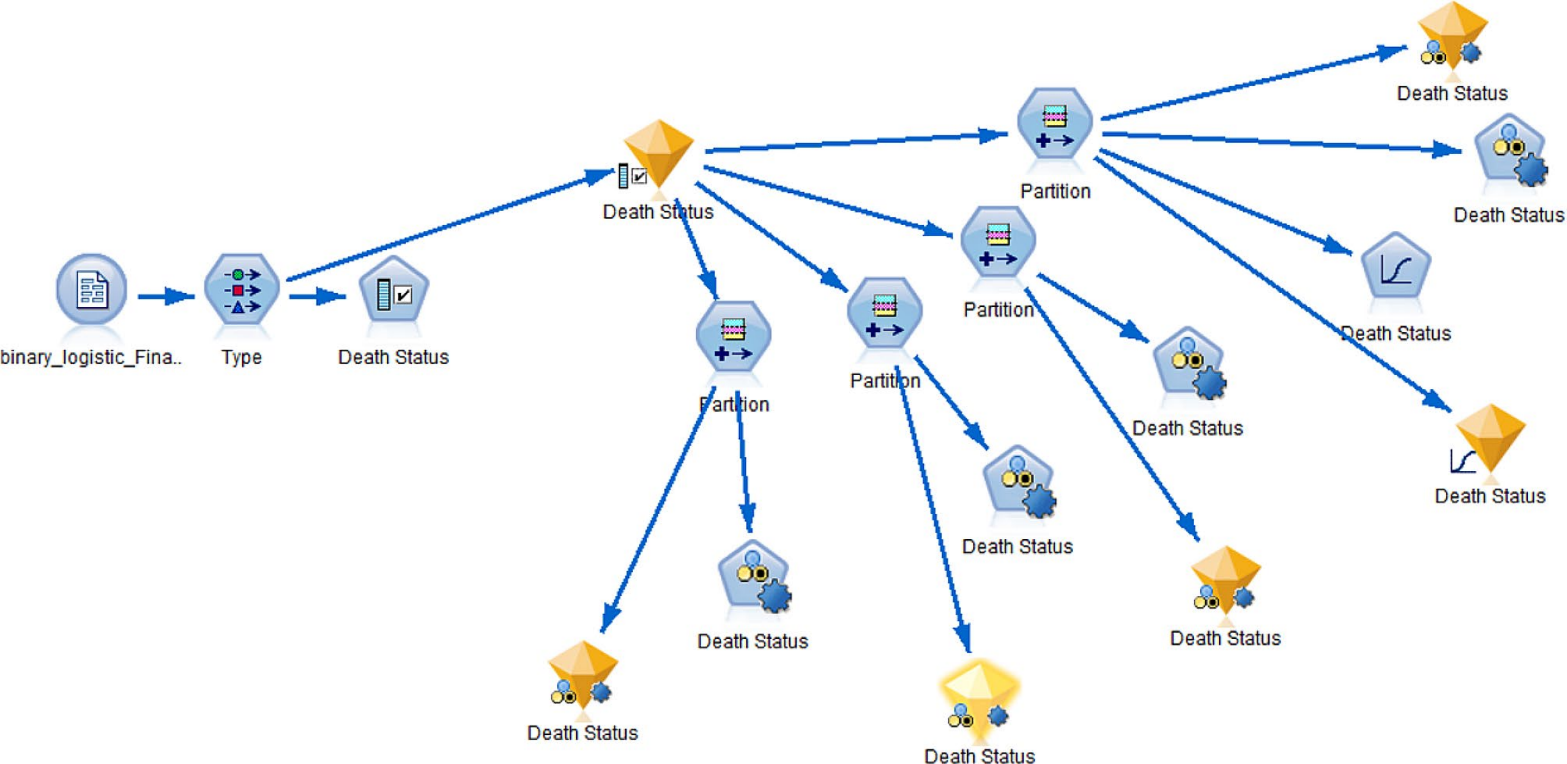
the graphical display of the analysis procedure

Influence of different training and testing ratios on the performance of the given ML models checked. The study divide the dataset into training and testing datasets for the performance assessment of models. Popular statistical indicators are employed to evaluate the predictive capability of the models under different training and testing ratios. After using feature selection, the study considered 90%/10%, 80%/20%, 70%/30%, and 60%/40% train/test partitions. The partition procedure shows in the following figure (Fig. 3). Comparison criteria are lift and AUC.

## Result

The study involved 1520 individuals, with an average age of 24 years and an average weight of 42 kg. Their average initial systolic and diastolic blood pressure measurements were 107 and 66 respectively. The average oxygen saturation (SPO2) level was also found to be 95%. The average ejection fraction was 59%. On average, patients had to wait 481 days for their surgical procedures (Table 1).

**Table 1:** Descriptive analysis for continuous variables

Variable	Definition	Range	Average	Standard deviation
Age	Age of cardiac patient	84	24.44	18.66
Weight in KG	Weight of cardiac patient	157	42.48	22.36
Systolic Blood Pressure	Systolic Blood Pressure	140	107.09	13.66
Diastolic blood pressure	Diastolic Blood Pressure	96	66.88	10.92
Pulse rate	Pulse rate	148	84.48	19.12
SPO2	Oxygen saturation	58	94.73	3.95
INR	International normalised ratio	10.30	2.63	0.75
Ejection fraction	Post-surgery ejection fraction	47	59.05	6.78
ICU stay	Post-surgery intensive care unit stay	11	1.91	1.09
Duration of time in cardiac center after surgery	Post length of cardiac center stay in day	42	7.46	4.72
Waiting time in day	Time to surgery in day	3139.0	480.56	600.40
Hemoglobin value		20.2	12.30	1.68
Creatinine value		2.40	0.70	0.28

Among 1520 patients included in the study, 122 individuals (8% of the total) were died. Among the patients who underwent cardiac surgery, there were 842 females, 692 patients from rural areas, valvular heart disease cases (874), congenital cases (593), and other cases (53). There were 145 patients with a family history of heart disease, patients with heart failure (20), smokers (4), patients with normal sinus rhythm (49), patients who had undergone multiple surgeries (25), and 177 patients with joint heart conditions.

Based on the chi-square association, there was a significant correlation found between the outcome status (Death or Alive) and predictor variables including heart disease type, family history, rheumatic disease status, heart failure status, and NYHA class (Table 2).

**Table 2:** The chi-square tests of association among categorical variables and death status among participants under investigation

Predictors/categories		Status		Total	Pearson Chi-Square	*P* value
		Alive	Death			
Gender	Female	780	62	842	1.124	0.289
	Male	618	60	678		
Residence	Rural	640	52	692	0.451	0.502
	Urban	758	70	828		
Heart disease type	Congenital	575	18	593	39.734	< 0.001
	Other	42	11	53		
	Valve Disease	781	93	874		
Rheumatic disease status	Non Rheumatic	372	15	387	12.115	0.001
	Rheumatic	1026	107	1133		
Family History	NO	1273	102	1375	7.221	0.007
	Yes	125	20	145		
NYHA class	II	543	26	569	15.077	0.001
	III	769	88	857		
	IV	86	8	94		
Baseline diabetes status	NO	1385	121	1506	**	^**^
	Yes	13	1	14		
Pulmonary hypertension status	NO	1347	115	1462	1.335	0.248
	Yes	51	7	58		
Stroke status	NO	1397	119	1516	**	^**^
	Yes	1	3	4		
Heart Failure Status	NO	1391	109	1500	89.115	< 0.001
	Yes	7	13	20		
Smoking status	NO	1397	119	1516	**	^**^
	Yes	1	3	4		
Number of surgery	More than one	24	1	25	**	^**^
	One	1374	121	1495		
Joint heart case	Combination	161	16	177	0.279	0.598
	Single	1237	106	1343		
Cardiac rhythm	Atrial fibrillation	1351	120	1471	**	^**^
	Normal sinus rhythm	47	2	49		

** shows lack of chi-square assumptions

logistic regression machine learning algorithm was preferable through AUC on 90/10 partition than others expressed from testing data. Finally, a logistic regression algorithm was implemented to analyze other than CHID, QUEST, Bayesian network, Neural network, C5, Decision list, Discriminant, and C&R machine learning algorithms. The model with an AUC value approach to one is preferable and reasonable to choose. As shown below, the AUC value of logistic regression under 90%/10% partition for cardiac patients was 0.961, greater than other algorithms (Table 3). In addition, based on the lift value of 3.33, it can be concluded that the logistic regression algorithm was performing well and providing substantial improvement over random selection. Lift value greater than 1(one) in general considered as a good fit. The higher lift value suggests that the model has a good ability to discriminate between the target and non-target outcomes, and it can be considered a crucial tool for making predictions and driving decision-making processes.

**Table 3:** area under the curve performance of different algorithms for cardiac patient’s surgery outcome

Model	AUC value in train/test partition			
	90%/10%	80%/20%	70%/30%	60%/40%
Logistic regression	0.961	0.946	0.859	0.864
Discriminant	0.948	0.941	0.855	0.854
Neural Net	0.941	0.951	0.868	0.865
CHAID	0.896	0.819	0.832	0.719
Decision List	0.86	0.831	0.714	0.746
Bayesian Network	0.836	0.74	0.619	0.638
C5	0.845	0.864	0.712	0.782
C&R Tree	0.851	0.751	0.712	0.702
Quest	0.74	0.749	0.5	0.701

**Fig. 4:**
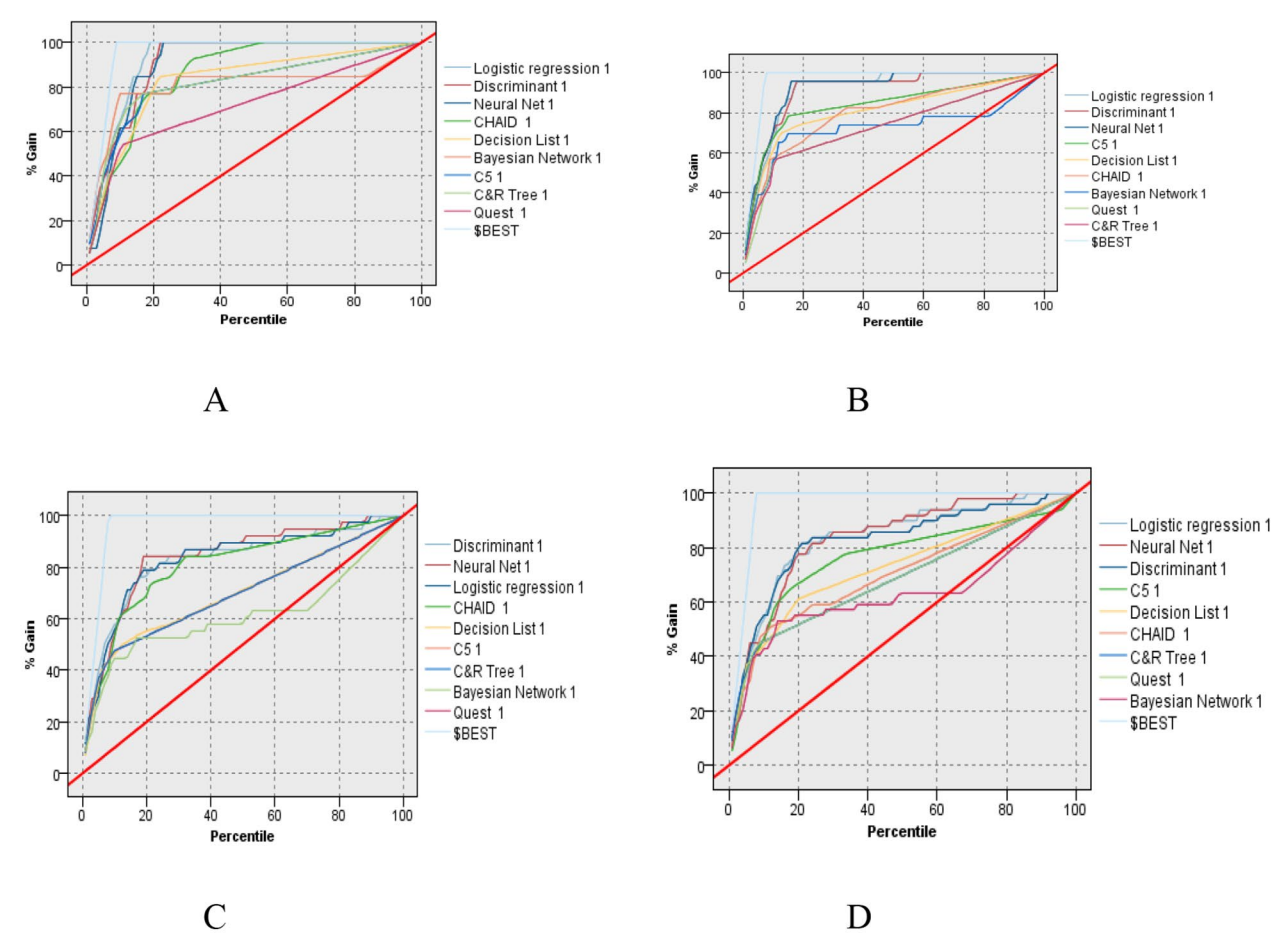
(**A**) 90/10 partition Gain graph. (**B**) 80/20 partition Gain graph. (**C**) 70/30 partition Gain graph. (**D**) 60/40 partition Gain graph

In addition to the global comparison method, the gain curve shows the curve approaches the best curve or the fastest to 100% is considered best (see Fig. 4A), and again logistics is a good fit for cardiac patients’ surgery outcomes since it is approved graphically too (Fig. 4(A-D)).

**Fig. 5:**
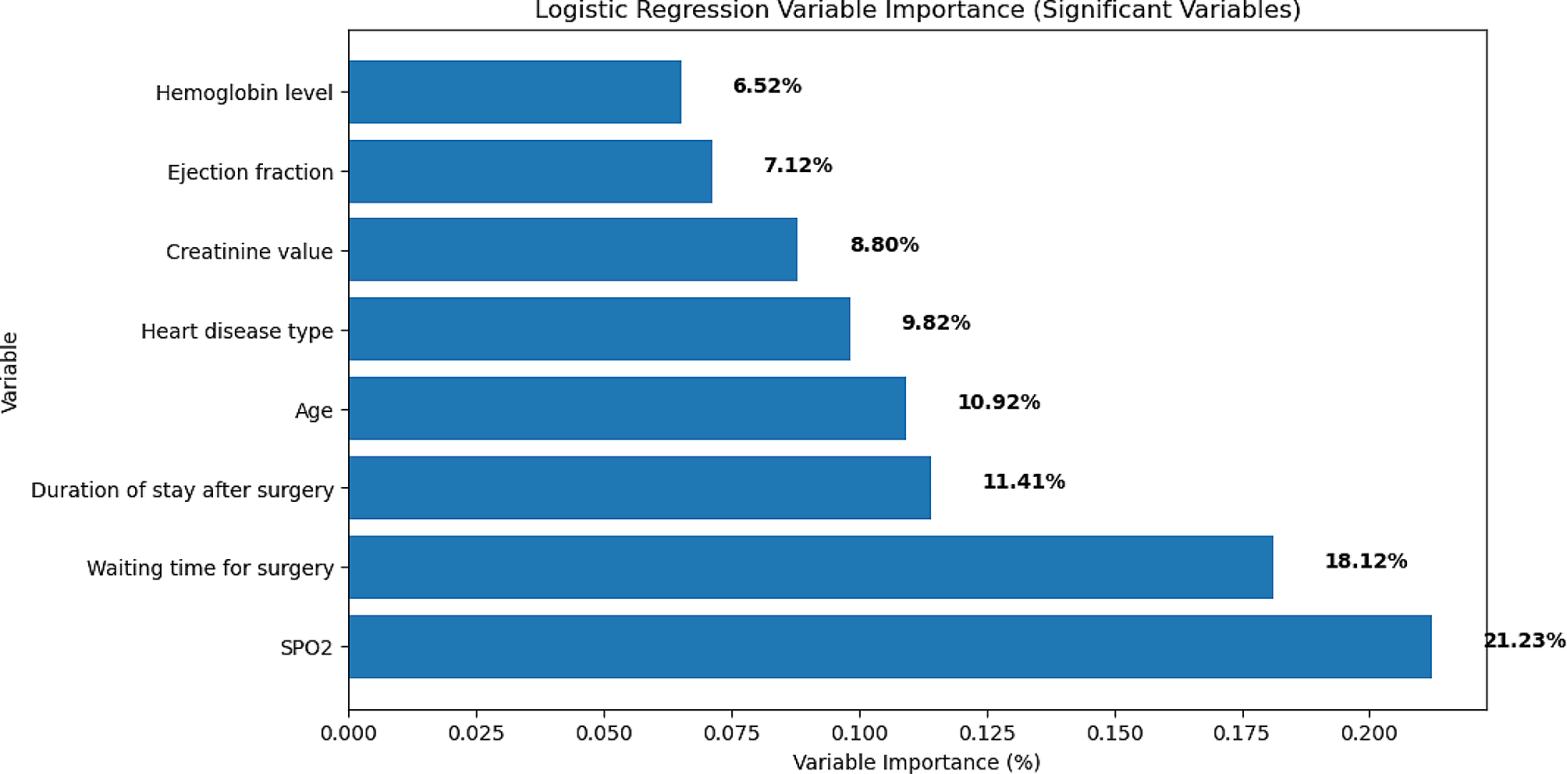
The relative predictor importance

The importance of the logistic regression algorithm over other algorithms is in addition to predictor relative importance; it gives us a coefficient for interpretation. For cardiac surgery patients’ outcome prediction, from all predictors in the final model, the most important predictors were SPO2, creatinine value, age, waiting time for surgery, length of cardiac center stays after surgery, hemoglobin value, ejection fraction, and heart disease status. From logistic algorithm, SPO2 contributes approximately 21.23% to the model, age contributes 10.9%, time to surgery waiting time contributes approximately 18.12%, length of cardiac center stays after surgery values 11.14%, and creatinine value contributes 8.80%, heart disease type impacts 9.82%, ejection fraction 7.12% and hemoglobin value contributes approximately 6.52% (Fig. 5).

From advanced modelling results, age, ejection fraction, SPO2, length of cardiac center leave day, waiting time, haemoglobin value, and creatinine value were significant predictors of death. When age increased by one year, the expected odds for the occurrence of death among cardiac patients was increased by 80.3%. For a single unit rise of SPO2, the expected odds for the occurrence of death among cardiac patients was decreased by 0.355 time as compare to the other groups, given other covariates constant. In another way, for a single unit rise of SPO2, the expected odds of occurrence of death were decreased by 64.5% given the other covariates constant. About ejection fraction, for a single unit increasement, the expected odds of occurrence of death were decreased by 27.5%. On the other hand, as patient’s waiting time after surgery increased by one day, the expected odds for occurrence of death was 2.3 times greater as compared with the previous day. For a single unit increasement of hemoglobin value, the expected odds of occurrence of death for a cardiac patient was decreased by 33%, given the other conditions constant. When creatinine value increased by a single unit, the expected odds of occurrence of death were also increased by 58% (Table 4).

**Table 4:** Multivariable binary logistic regression result

Predictors	B (Coefficient)	OR(CI)	*P* value
Heart Disease Type			0.197
Other	-0.672	0.511(0.185, 1.406)	0.193
Valvular	-0.692	0.501(0.174, 1.443)	0.200
Congenital (Ref)			
Rheumatic disease Status (Rheumatic type)	-0.350	0.704(0.259, 1.919)	0.493
Rheumatic Disease Status (Non-romantic) (Ref)			
NYHA Class			0.518
III	0.004	1.004(0.355, 2.837)	0.995
IV	0.319	1.375(0.548, 3.452)	0.497
II (ref)			
Age	0.590	1.803(1.285, 2.530)	0.001
Weight	0.033	1.034(0.683, 1.565)	0.875
SPO2	-1.035	0.355(0.283, 0.445)	< 0.001
INR	0.044	1.045(0.843, 1.296)	0.689
Ejection Fraction	-0.322	0.725(0.576, 0.911)	0.006
Length of cardiac center leave Day	-0.459	0.632(0.487, 0.819)	0.001
waiting time for surgery	0.858	2.358(1.845, 3.014)	< 0.001
Hemoglobin Value	-0.252	0.777(0.635, 0.952)	0.015
Creatinine Value	0.352	1.423(1.140, 1.775)	0.002

## Discussion

The objective of the research was to identify the factors that contribute to the likelihood of post-surgery death in cardiac patients. The study found several significant predictors of death outcome, including waiting time, hemoglobin value, creatinine value, ejection fraction, duration of cardiac center stays after surgery, saturated oxygen, and age.

The death prevalence due to cardiac disease among heart patients included in the study was 122(8%). The finding is consistent with other previously conducted researches [31–34].

Age is one of the significant predictors [33–37]. This alignment further supports the investigation’s results. As patients grow older, the likelihood of experiencing complications and comorbidities increases. Moreover, the effectiveness of treatments tends to decrease with advancing age. The physiological aging of the heart plays a significant role in the development and occurrence of cardiac issues in older individuals, primarily due to heightened inflammation and oxidative stress [38].

Creatinine value is crucial in predicting death [36, 39, 40]. Given its significance, the role of creatinine as a powerful predictor has been incorporated into various mortality risk assessment tools currently used for patients undergoing cardiac surgery [41–43]. Creatinine test is conducted to evaluate the functioning of the kidneys, as they are responsible for removing creatinine from the body. If the kidneys are not functioning properly, the level of creatinine in the blood will increase due to the kidneys’ reduced ability to clear creatinine effectively. This elevated creatinine level indicates damaged kidney function.

Saturated oxygen were the main predictors for predicting death [44]. Low saturated oxygen levels have been associated with an increased risk of adverse cardiac events and mortality in heart patients. Insufficient oxygen supply to the heart can exacerbate existing cardiac conditions, contribute to the development of complications, and potentially lead to fatal outcomes.

The lower ejection fraction was the responsible predictor of death [37, 39, 45–47]. A low ejection fraction indicates that the heart is unable to pump enough blood, which can ultimately lead to heart failure. In addition, it may lead to shortness of breath. This could potentially contribute to increase the chance of death outcome.

Hemoglobin result was related with worse outcome [34]. Anemia occurs when the hemoglobin levels in the blood are low. This condition can negatively impact cardiac function in multiple ways. Anemia cases stress on the heart by causing increased heart rate and it can result in reduced blood flow to the kidneys.

The average waiting time for surgery was 481 days. According to a statement from the Ethiopian Ministry of Health, the figure presented underestimates the true situation regarding cardiac surgery patients and their waiting times. The average waiting time mentioned in the Ministry statement is seven years. However, the study results indicate that this estimation is lower than the actual waiting times. This is because the study only took into account the waiting time after referral for cardiac surgery admission. In reality, patients may have to wait for a considerable amount of time for free surgery services, and if they become too ill, they may choose to opt for private surgery service, which is provided immediately. The urgency level might also affect the figure of waiting time. These factors undermine the accuracy of the waiting time results. The duration of waiting time to get surgery was found to be highly correlated with and served as the most significant predictor of mortality among cardiac patients after surgery [48]. However, there are some papers that have suggested that the outcome of the surgery is not influenced by the waiting time [49, 50]. The difference might arise due to getting the availability of cardiac surgery service in a short period as compared to Ethiopia. It is important to note that Ethiopia has a limited number of cardiac centers, which serving a population of more than 126 million. This disparity in healthcare infrastructure could be a contributing factor.

## Conclusions and recommendations

To sum up, logistic regression machine learning was preferable to other algorithms. Ejection fraction, age, saturated oxygen, duration of cardiac center stays after surgery, waiting time, hemoglobin value, and creatinine value were responsible predictors of death outcome.

According to worldometr figure, Ethiopia has about more than 126 million people, making it the 11th most populated country globally and the 2nd most populous in Africa [51], a lot of health investment in the field of cardiac is a future task. Serving this total population in a very resource limited centers might be also unrealistic.

For ministry of health, Health Bureaus, health institutes, national and local Governments, Universities, local and international organizations, NGO’s, lack of cardiac surgery service in Ethiopia is a serious concern as such, to reduce the waiting time, the study suggest to increase health facilities like cardiac centers, hospitals or institutes which serve as cardiac surgery in Ethiopia for at least 10 regions in a short period. So, the allocation of required budget and supplies through collaboration with other partners or stakeholders are timely. Those stakeholders are also required to design or formulate sustainable income generating activities of those centers. In addition, universities are expected to graduate the required number of cardiac specialists too. For health professionals and health facilities, the study recommended to give special attention for cardiac patients with lower saturated oxygen, higher age, patients with elongated waiting time to surgery, lower ejection fraction value, lower hemoglobin value and higher creatinine value. The study also recommends Ministry of Health and Federal Government to established and realized “Cardiovascular Institute”.

The findings of this study will be helpful for Ministry of Health, clinicians, and policymakers to develop policies to reduce the burden of cardiac disease in the country. Therefore, this study provided current situation of cardiac patients’ burden after surgery.

### Limitation of current study:

This study limitations is that there was limited information about the current burden status of cardiac patients in each cardiac center in Ethiopia. Hence, the current study recommends future researches to conduct research including important information related to the burden of cardiac patients after surgery.

## Data Availability

The data used in the current study is available under the corresponding author and can be attached on request.

## Abbreviations

NYHA: New York Health Association
SPO2: Oxygen Saturated
INR: International Normalised Ratio
AUC: Area Under the Curve
CHAID: Chi square Automatic Interaction Detection
QUEST: Quaternion Estimator
C&R: Classification and Regression
ML: Machine Learning
